# High‐Yield Extruded Nanovesicles From Adipose Stem Cells Promote High‐Quality Healing of Diabetic Wound Through WNT/β‐Catenin Pathway Activation

**DOI:** 10.1111/jcmm.70877

**Published:** 2025-10-07

**Authors:** Tonghao Yao, Liangliang Liu, Yibo Miao, Xinxin Li, Ying Yu, Rongyao Sun, Yining Zhang, Luping Cui, Xu Ma

**Affiliations:** ^1^ Department of Plastic and Aesthetic Surgery The Second Affiliated Hospital of Harbin Medical University Harbin China; ^2^ Department of General Surgery Department The Second Affiliated Hospital of Harbin Medical University Harbin China

**Keywords:** ADSCs, diabetic wound, extracellular vesicles, fibroblasts, Wnt/β‐catenin

## Abstract

Diabetes is a significant global chronic disease characterised by elevated mortality and disability rates due to persistent infections resulting from refractory wounds. Currently, effective treatment strategies are lacking. Adipose‐derived stem cell extracellular vesicles (ADSC‐EVs) have been shown to promote skin wound healing; however, their clinical application is impeded by low yield and heterogeneity. We successfully isolated high‐yield extruded nanovesicles from adipose stem cells (ADSC‐NVs), achieving yields over 30 times greater than those of ADSC‐EVs while maintaining similar mor‐phological characteristics. Our findings indicate that ADSC‐NVs exhibit a dose‐dependent en‐hancement of proliferation and migration in primary human dermal fibroblasts (HDF) in vitro. Notably, the expression levels of proliferating cell nuclear antigen (PCNA), collagen type I (COL‐I) and collagen type III (COL‐III) were significantly upregulated in HDF following treatment with ADSC‐NVs. RNA‐seq analysis further revealed that the differentially expressed genes (DEGs) shared between the ADSC‐NVs group and control group were predominantly enriched in the Wnt signalling pathway. Consistently, ADSC‐NVs facilitate efficient diabetic wound healing while promoting proliferation and inhibiting inflammation via the Wnt/β‐catenin signalling pathway. In summary, high‐yield ADSC‐NVs represent a promising alternative to ADSC‐EVs for enhancing diabetic wound healing, providing novel insights and methodologies for improving therapeutic outcomes.

## 1 Introduction

Diabetes is a significant global chronic illness, associated with elevated rates of mortality and disability [1]. A prevalent complication of diabetes is the occurrence of non‐healing wounds. These persistent diabetic ulcers not only lead to systemic infections but also contribute to prolonged hospitalisation, diminished quality of life, and an increased burden of disability [2, 3, 4, 5]. The normal wound healing process encompasses several stages: blood clot formation, inflammation, tissue regeneration (including re‐epithelialization and granulation tissue formation), followed by remodelling and repair [6, 7, 8]. However, diabetic wounds frequently stagnate at these phases, impeding the natural healing trajectory and resulting in chronic wound development [9]. Wound recovery is a complex and coordinated process involving multiple stages in which fibroblasts play a pivotal role by signalling other essential cell types to seal and fill the wound area [10, 11].

In recent years, there has been a significant increase in both research and interest surrounding adipose‐derived stem cells (ADSCs), particularly regarding their application in cell therapy for skin damage repair. As pluripotent cells, ADSCs possess the capacity to differentiate into various cell types [12, 13]. Compared to mesenchymal stem cells (MSCs), ADSCs offer several advantages, including greater availability, reduced invasiveness, and the absence of ethical constraints [14, 15]. Extracellular vesicles (EVs), specifically exosomes ranging from 40 to 160 nm in size, are essential for intercellular and tissue communication through the transfer of genetic and molecular materials [16]. In contrast to traditional cell therapy, EVs as a form of cell‐free therapy present numerous benefits such as enhanced stability, lower immunogenicity, fewer ethical concerns, and diminished risk of malignant transformation [17, 18]. Recent studies have highlighted the importance of EVs in wound repair; EVs derived from MSCs play a crucial role by stimulating re‐epithelialization, enhancing collagen maturation, and minimising scarring in acute skin injuries. The ability of EVs to promote angiogenesis and mitigate inflammation is vital for facilitating this process—traits inherited from their parent cells [19]. However, limitations exist that hinder the widespread clinical application of MSC‐derived exosomes. The production of these EVs necessitates large quantities of consistently expanded MSC populations that are prone to cellular senescence along with variable differentiation potential and passage‐specific alterations—factors contributing to decreased stability and reproducibility of exosome yields [20]. Typically yielding only 0.1 mg per day per 10^6^ cultured MSCs as determined by membrane protein concentration via Bradford assay methods; this low yield combined with time‐consuming purification processes restrict its potential applications in clinical settings [21].

Recently, to address the production limitations of extracellular vesicles (EVs), engineered exosomes known as cell nanovesicles (NVs) have been developed as mimics of EVs [22]. NVs are generated by continuously forcing cells through micropores and nanopores, whereas EVs are naturally secreted by cells. Notably, for an equivalent number of cells, the yield of NVs is approximately 200 times greater than that of EVs, with no significant differences observed in size, morphology, or protein markers. Furthermore, analyses of protein and RNA cargo indicate that NVs possess biological functions to a certain extent and can serve as viable substitutes for EVs [23, 24, 25]. NVs derived from embryonic stem cells were initially designed to act as alternatives to EVs in promoting the proliferation of human dermal fibroblasts [22].

In this study, we generated NVs from ADSCs for the purpose of chronic skin wound healing. Initially, we compared the effects of ADSC‐EVs on isogenic primary human dermal fibroblasts. Differentially expressed mRNAs were identified through high‐throughput sequencing of human skin fibroblasts treated with ADSC‐NVs. Notably, ADSC‐NVs induce proliferation, collagen metabolism, and migration in human dermal fibroblasts via the WNT/β‐catenin signalling pathway. Concurrently, we established a diabetic wound model by administering streptozotocin to C57BL/6 mice to assess both the rate of healing and the quality of wound tissue while further exploring the beneficial molecular mechanisms associated with ADSC‐NVs. In comparison to ADSC‐EVs, ADSC‐NVs represent a viable alternative and offer a promising strategy for cell‐free therapeutic approaches.

## 2 Materials and Methods

### 2.1 Cell Culture

**FIGURE 1:**
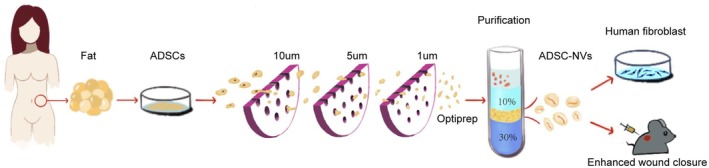
Schematic showing the preparation of ADSC‐NVs. To achieve high‐quality wound healing by promoting the proliferation and migration of HDF.

The study conducted at the Second Affiliated Hospital of Harbin Medical University in 2023 involved the recruitment of women aged 20 to 50 for liposuction surgery. The Ethics Committee of Harbin Medical University approved the study prior to its initiation. Tissue samples of subcutaneous fat and skin were collected from the participants' abdomens. Informed consent was obtained from each participant before establishing ADSCs through primary culture of human subcutaneous fat. These cells were subsequently suspended in Dulbecco's Modified Eagle Medium, supplemented with 10% fetal bovine serum and 100 IU penicillin/100 mg/mL streptomycin (Figure 1). The cells were cultured at 37°C in a humidified atmosphere containing 5% CO_2_. Additionally, primary cultures of HDF were performed using a similar methodology as that employed for ADSCs.

### 2.2 Characterisation of ADSCs and Induction of Osteoblastic and Adipogenic Differentiation of ADSCs

To confirm the successful extraction and specific characteristics of the isolated ADSCs, we employed flow cytometry. The identity of the cell type was determined by the presence or absence of specific cell surface markers. ADSCs are characterised by the expression of CD29 and CD90 proteins, along with the lack of CD14 and CD45 proteins. Cultured ADSCs were isolated and suspended in phosphate‐buffered saline (PBS) supplemented with 2% fetal bovine serum (FBS), achieving a cell density of 2.0 × 10^7 cells/mL. Antibodies specific to CD29 (BioLegend, catalogue number 303003), CD90 (BioLegend, catalogue number 328107), CD45 (BioLegend, catalogue number 305005) and CD14 (BioLegend, catalogue number 325603) were subsequently added to the cell suspension, which was maintained on ice for 30 min. The cell solution was then washed by centrifugation at 840 g for 5 minutes; after discarding the supernatant, the pellet was resuspended in PBS with 2% FBS before undergoing an additional centrifugation at 840 g for 5 minutes. Following this procedure, the cell solutions were transferred to test tubes and analysed using an LSRFortessa flow cytometer (BD Biosciences). After reaching approximately 80% to 90% confluence following passage three, growth medium was switched to either osteoblast induction medium or adipogenesis induction medium (both from OriCell, China). On Day 21 post‐induction, Alizarin Red S staining (Solarbio, China) was performed to evaluate osteogenic differentiation; conversely, Oil Red O staining (Solarbio, China) was conducted on Day 14 post‐induction to assess lipid droplet formation. Microscopic examination of stained samples utilised a microscope equipped with a camera system from Leica Microsystems GmbH in Germany for image capture.

### 2.3 Isolation and Analysis of ADSC‐EVs and ADSC‐NVs

ADSCs at passages 3 to 5 were utilised for the generation of NVs. Upon reaching over 90% confluence, the cells were detached using a solution of 2 mM ethylenediaminetetraacetic acid (EDTA) and subsequently resuspended in 1 mL of PBS. To ensure effective separation of nanovesicles from larger cellular components, the cell suspension underwent five rounds of filtration through filters with progressively smaller pore sizes: 10, 5 and finally 1 μm, utilising a small extruder from Avanti Polar Lipids. Following this filtration process, centrifugation was performed at 1000 g for 5 min; this step was critical for removing intact cells and larger debris that could interfere with subsequent analyses. The isolation of nanovesicles was then achieved via bottom‐up iodixanol density gradient ultracentrifugation. In this context, it is noteworthy that the working solution of Opti‐prep (AXIS‐SHIELD) was prepared as a discontinuous iodixanol gradient consisting specifically of a 30%–10% Opti‐prep solution within an ultracentrifuge tube. After subjecting the prepared solution to extrusion, it was layered over the Opti‐prep working solution and ultracentrifuged at high speed—100,000 g—for seventy minutes. The nanovesicles ultimately accumulated between layers corresponding to concentrations ranging from 10% to 30% in the Opti‐prep working solution. To conclude the process, final nanovesicle particles were resuspended in PBS for subsequent analysis or application.

The size of purified EVs and NVs was measured using the NanoSight LM10 nanoparticle tracking system from Malvern Instruments. Proteins TSG101, CD81 and calnexin were identified via Western blotting (Abcam, USA). The PKH26 red fluorescent cell labelling kit was utilised to label ADSC‐NVs in accordance with the manufacturer's guidelines (Solarbio, China). Protein concentration in exosomes was quantified using the BCA protein assay kit from Beyotime, China. The ultrastructure of extracellular vesicles was examined through transmission electron microscopy (TEM) utilising a Libra 120 instrument from Zeiss.

### 2.4 Human Dermal Fibroblasts (HDF) Treatment

HDF (5 × 10^6^ cell) treated with ADSC‐EVs, ADSC‐NVs (50 μg/mL) or PBS (control). Next, we treat the cells with the Wnt/β‐catenin inhibitor XAV939 (40 nM, M1796, AbMole, USA), and the cells were harvested after 24 h of treatment and used for subsequent experiments.

### 2.5 RNA‐Sep

All reagents for cell culture were obtained from Gibco (New York, USA), with the culture medium refreshed every other day. HDF were divided into two experimental groups: one treated with phosphate‐buffered saline (PBS) and the other treated with ADSC‐NVs.

Samples were collected from the HDF + PBS and HDF + ADSC‐NVs groups (*n* = 4). Following two washes with PBS, RNA was extracted. RNA sequencing was performed by Shanghai Biotechnology Corporation (Shanghai, China) using a second‐generation Illumina high‐throughput sequencing platform, in accordance with the TRIZOL method. A total of 34,745 genes were detected and compared between the HDF + PBS and HDF + ADSC‐NVs groups. Transcripts were analysed using DESeq2 (adjusted *p*‐value < 0.05), identifying target genes with ∣log2(fold change)∣ > 1.

### 2.6 RNA Extraction and Real‐Time PCR

Total RNA was extracted from both cells and tissues following treatment protocols using RNAiso Plus (TaKaRa Biotechnology, Shiga, Japan), in accordance with the manufacturer's guidelines. The isolated RNA was subsequently converted into complementary DNA (cDNA). Thereafter, mRNA expression levels were assessed through real‐time PCR utilising an ABI Prism 7500 Sequence Detection System (ABI, CA, USA) with SYBR Premix ExTaq (TaKaRa Biotechnology).

### 2.7 Western Blot Analysis

ADSC‐EVs, ADSC‐NVs, cells, or tissues were lysed using RIPA buffer (Beyotime Biotechnology, P0013B) containing 1% PMSF (Aladdin, P105539), and the supernatants of the lysates were used for subsequent Western blot analysis. Protein samples were separated using a 10% SDS‐PAGE gel and subsequently transferred to a PVDF membrane (Millipore, IPVH00010). Following blocking with 5% skim milk in TBST for 2 h, the membrane was subjected to overnight incubation at 4°C with various primary antibodies including CD81 (Abways, CY5742), TSG101 (Abways, CY5985), Calnexin (Abways, CY5839), GAPDH (Abcam, ab181602), Type I collagen (COL‐I) (Proteintech, 66761‐1‐lg), Type III collagen (COL‐III) (Proteintech, 22734‐1‐AP), α‐SMA (Abcam, ab5694), PCNA (Abways, AB0051), Wnt2b (Abways, CY8167), beta‐catenin (Cell Signalling Technology, 8480S) and beta Actin (Abways, AB0035). On the subsequent day, the membrane underwent washing with TBST and was subsequently incubated with HRP‐conjugated goat anti‐rabbit antibody (Abcam) for a two‐hour period at ambient temperature. Ultimately, the bands were visualised utilising ECL blotting detection reagent (Servicebio, G2020).

### 2.8 Mmunocytochemistry

To assess cell proliferation, samples were treated with Ki67 antibody (Abcam, USA), α‐SMA (Abcam, ab5694)overnight at 4°C. Following three washes with phosphate‐buffered saline (PBS), the specimens were incubated with secondary antibodies (Aspen, China) for 1 h at ambient temperature. Subsequently, microscopic images were captured and analysed using the ImageJ software platform.

### 2.9 Cell Proliferation Assay

An equivalent initial number of HDF was seeded in a 96‐well culture plate and allowed to proliferate for 24 h. After treatment with nanovesicles at varying concentrations for an additional 24 h, 10 μL of CCK‐8 solution was added to each well. The culture plate was then incubated for 2 h, and absorbance at 450 nm was measured using a microplate reader (Beckman Coulter). Cell viability of treated versus untreated fibroblasts was subsequently compared. Cell proliferation was assessed using the BeyoClick EdU‐594 Cell Proliferation Detection Kit (Beyotime, Shanghai, China) according to the manufacturer's protocol. Images were captured with an inverted fluorescence microscope (Leica Microsystems). Red fluorescence indicated positive cells, while Hoechst‐stained nuclei exhibited blue fluorescence.

### 2.10 Cell Migration

In a 6‐well plate, introduce 2 × 10^5^ HDF, and gently scrape the smooth edge using a 200 μL pipette tip. Following this, add 500 μL of serum‐free DMEM medium supplemented with extracellular vesicles(50 μg/mL ADSC‐EVs, ADSC‐NVs) or PBS to each well. Photographs were taken with a Leica inverted microscope after 0, 12 and 24 h, and the migration area was calculated after 0, 12 and 24 h (a Leica microscope was used for the camera). Likewise, add exosomes to the bottom of the transwell plate. Place HDF in the upper chamber of the Transwell containing serum‐free medium. After a 24‐h period, the epithelial cells were eliminated using a cotton swab and subsequently rinsed. We applied 0.1% crystal violet for staining and captured images utilising a Lecka inverted microscope.

### 2.11 Diabetic Wound Healing Evaluation

Eight‐week‐old C57BL/6 mice were procured from the Second Affiliated Hospital of Harbin Medical University, following the green light from the Institutional Animal Care and Use Committee (IACUC). This study is reported in accordance with ARRIVE guidelines. All experiments were performed in accordance with relevant guidelines and regulations. These mice were subjected to a high‐fat diet consisting of 45% fat for a duration of 5 weeks. After a 12‐h fasting period, the mice were administered an intraperitoneal injection of streptozotocin (40 mg/kg, 0.1 M citrate‐buffered saline, pH 4.5) to induce diabetes mellitus (DM). Blood glucose monitoring was carried out using an Accu‐Check Active blood glucose metre (Roche, Lyon, France), and mice were deemed diabetic if their blood glucose levels surpassed 16.7 mmol/L. To ensure the stability of the DM model, the diabetic mice continued on the high‐fat diet for an additional 2 weeks, with blood glucose levels confirmed prior to any wound induction. Anaesthesia was facilitated using sodium pentobarbital (Sigma‐Aldrich) (1%, 50 mg/kg). A full‐thickness circular skin wound measuring 6 mm in diameter was created on the dorsal surface of each mouse. The diabetic mice were then Using a random number generator, they were randomly divided three treatment groups for subcutaneous injections: ADSC‐EVs (DW + EVs), ADSC‐NVs (DW + NVs) (50 μg in 100 μL PBS), or a control group receiving 100 μL PBS (DW). Each group of mice consists of 8 individuals to ensure the accuracy of the experiment. Injections were administered at four spaced locations around the perimeter of the wound. The control group, designated as Ctrl, received identical wound treatments while maintained on a standard diet. Photographs documenting the wounds were taken on Days 0, 3, 7 and 14 post‐surgery to evaluate healing progress, with wound sizes measured using ImageJ software. Mice were humanely euthanized by intraperitoneal injection of sodium pentobarbital (150 mg/kg), ensuring deep anaesthesia and followed by death.

### 2.12 Histology

Tissue specimens were preserved in a 4% paraformaldehyde solution prior to embedding in paraffin. Subsequently, the sections underwent overnight incubation at 4°C with primary antibodies specific to Ki67, COL‐I, COL‐III, IL‐6, or TGF‐β and were then treated with secondary antibodies (from Abcam) for 1 h at 37°C. Staining was performed using 3,3‐diaminobenzidine followed by counterstaining with haematoxylin. Additionally, Haematoxylin–eosin (H&E) and Masson's trichrome staining methods were employed. To assess the expression levels of COL‐I, COL‐III, TGF‐β, IL‐6 and VEGF mRNA in mouse tissues, quantitative reverse transcription PCR (qRT‐PCR) was conducted. Images were captured using a Leica microscope from which five random fields of view were selected.

### 2.13 Statistical Analysis

Data were analysed using GraphPad Prism 9 software and imagej, with results presented as mean ± standard deviation (SD). One‐way ANOVA and *t*‐tests were employed to compare two or more groups, establishing a significance threshold at *p* < 0.05.

## 3 Results

### 3.1 Isolation and Characterisation of ADSCs

**FIGURE 2:**
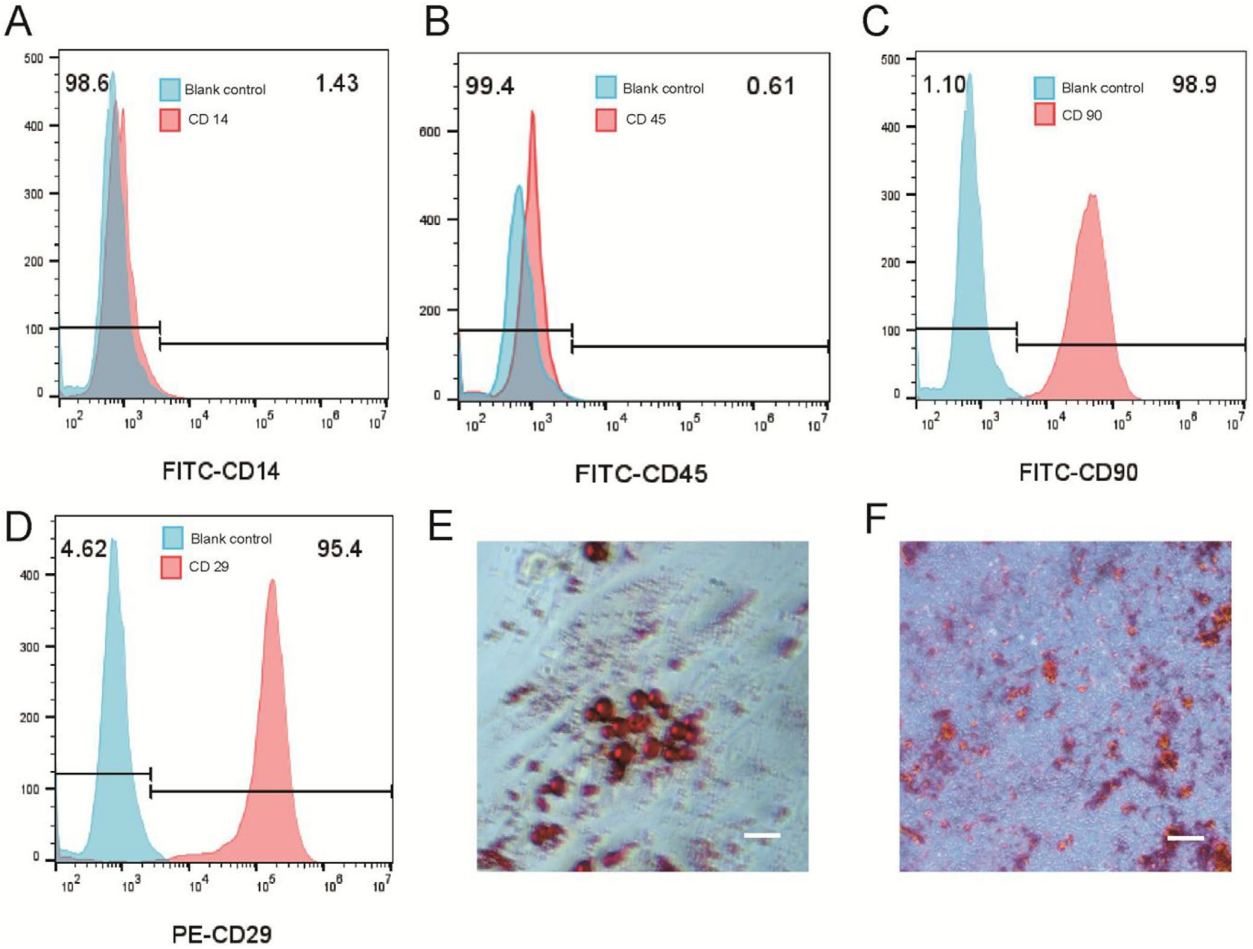
Characterisation of ADSCs. (A–D) Fow cytometry showed positive expression of CD29 and CD90, and negative expression of CD45 and CD14; (E) On day 14 of adipogenesis, fat vacuoles were stained orange by oil red O. (F) On day 21 of osteogenicinduction, redcalcium nodules were observed in the extracellularmatrix. Bars, 100 μm.

Throughout the duration of cell culture, it was observed that most adherent cells exhibited a spindle‐shaped morphology. By the time the cells had undergone three passages, their spindle shape became more pronounced and stable. Flow cytometry analysis of ADSCs demonstrated positive expression of specific stem cell markers CD29 and CD90, while showing negative expression for the haematopoietic marker CD45 and the leukocyte differentiation antigen CD14 (Figure 2A–D). After 21 days of osteogenic induction, ADSCs displayed a layered growth pattern lacking distinct structure, with red calcium nodules evident in the extracellular matrix as revealed by Alizarin Red S staining (Figure 2F). During adipogenic induction, small vacuoles with good light transmittance appeared in the cytoplasm of adipocytes by day four; by day fourteen, these vacuoles increased in number and size, with oil droplets exhibiting an orange‐red coloration typical of Oil Red O staining (Figure 2E). Collectively, these findings confirm the successful extraction and characterisation of ADSCs.

### 3.2 Characterisation of ADSC‐NVs

**FIGURE 3:**
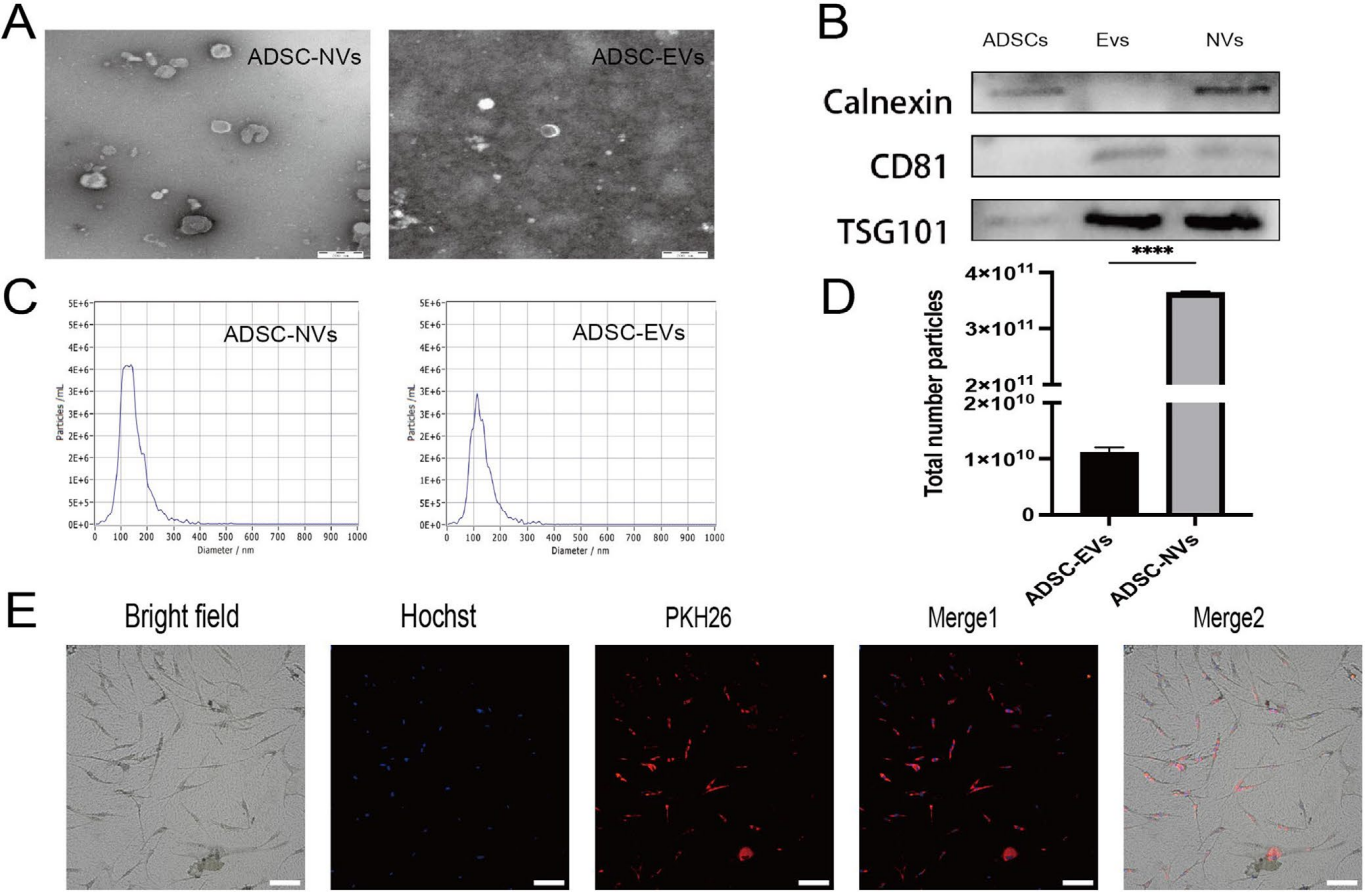
Characterisation of ADSC‐EVs and ADSC‐NVs. (A) Morphology observed under transmission electron microscope. Bars, 200 μm; (B) The expression of the ADSCs, ADSC‐EVs and ADSC‐NVs protein markers; (C) Particle size distribution; (D) Particle number; (E) Fluorescent microscopy analysis of PKH26‐labelled ADSC‐NVs internalisation by fibroblasts. ****p < 0.0001. Bars, 100 μm.

ADSC‐EVs were obtained through conventional differential ultracentrifugation, while ADSC‐NVs were produced using a sequential extrusion method that involved passing ADSCs through polycarbonate microfilters with progressively smaller pore sizes (10, 5 and 1 μm), followed by ultracentrifugation utilising an iodixanol density gradient. The characterisation of the isolated ADSC‐EVs and ADSC‐NVs included examination of their morphology, size distribution, and overall yield. Analysis via transmission electron microscopy (TEM) revealed that ADSC‐NVs consisted of spherical vesicles ranging from 100 to 150 nm in size, surrounded by a bilayer lipid membrane, resembling the structure of known ADSC‐EVs (Figure 3A). Nanoparticle tracking analysis (NTA) demonstrated comparable size distributions for both ADSC‐EVs and ADSC‐NVs, with average diameters falling within the range of 100 to 150 nm (ADSC‐EVs: 121 nm; ADSC‐NVs: 133.9 nm) (Figure 3C). The production efficiency for purified ADSC‐NVs and ADSC‐EVs derived from 1 × 10^7^ ADSCs was assessed by counting particle numbers; results indicated that approximately thirty times more ADSC‐NVs were produced than ADSC‐EVs, highlighting improved yield through the extrusion technique (Figure 3D). Western blot analysis confirmed the presence of calnexin in both ADSCs and ADSC‐NVs alongside detection of the transmembrane protein CD81 in both sample types. Tumour susceptibility gene‐101 (TSG101) was identified across all three sample types (Figure 3B). Additionally, red PKH26‐labelled content surrounding the nucleus provided evidence for internalisation of ADSC‐NVs by HDF (Figure 3E).

### 3.3 Promoted Fibroblast Proliferation and Migration by ADSC‐NVs In Vitro

**FIGURE 4:**
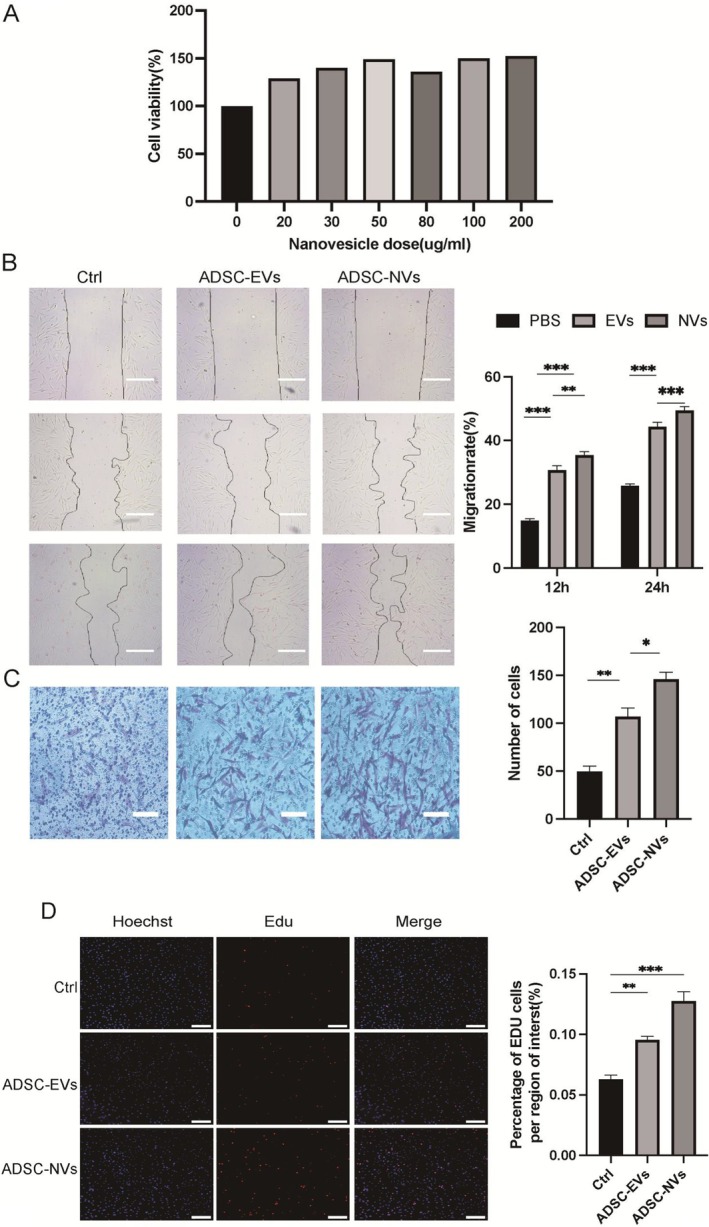
ADSC‐NVs significantly promote the proliferation and migration of human fibroblasts. (A) CCK‐8 analysis shows the proliferation results of fibroblasts at different concentrations of ADSC‐NVs; (B, C) the migration ability of HDF treated with ADSC‐NVs, measured by scratch and Transwell test assays; (D) the proliferation of cells by Edu assays. Data are represented as mean ± SD. *n* = 3. *p < 0.01, **p < 0.001, ***p < 0.0001. Scale bars, 100 μm.

ADSC‐NVs promote fibroblast proliferation and migration in vitro. To evaluate the capacity of ADSC‐NVs to stimulate cell proliferation, they were applied to syngeneic primary HDF at varying doses (0–200 μg/mL). The initial number of human skin fibroblasts was set at 2000 and seeded into 96‐well plates. After 24 h, the number of viable cells in samples treated with ADSC‐NVs was compared to that of untreated control cells using a CCK‐8 assay. Samples treated with concentrations of 20, 30, 50 and 80 μg/mL exhibited higher cell viability than untreated human skin fibroblasts (100%), showing values of 128.9, 140, 149.31 and 136.16%, respectively. Notably, samples treated with higher doses did not demonstrate significant differences from those treated with 50 mg/mL ADSC‐NVs (150% for both the treatments at 100 and 200 μg/mL). These findings suggest that elevated doses of nanovesicles do not enhance excessive proliferation in human skin fibroblasts and may even inhibit cell growth; thus, the optimal concentration for inducing proliferation is determined to be 50 μg/mL (Figure 4A).

To validate cell migration and proliferation in vitro, a scratch assay was performed. Cells within the scratch area were rinsed away using phosphate‐buffered saline (PBS), leaving behind the remaining cells, which were then exposed to either 50 μg/mL of ADSC‐NVs or ADSC‐EVs for a duration of 24 h. The reduction in scratch width correlated with increased cell activity in both migration and proliferation. Observations at 12 and 24 h revealed that samples treated with ADSC‐NVs exhibited the narrowest scratches, while untreated samples displayed the widest scratches. In conclusion, these findings suggest that administration of ADSC‐NVs enhances cell migration and proliferation (Figure 4B). This outcome was further corroborated through Transwell experiments, demonstrating a significant increase in fibroblast proliferation attributable to the presence of ADSC‐NVs (Figure 4C). Overall, ADSC‐NVs significantly promoted fibroblast proliferation (Figure 4D).

### 3.4 Studying the Mechanism by Which ADSC‐NVs Treatment Promotes Cell Proliferation Through RNA‐Seq Technology

**FIGURE 5:**
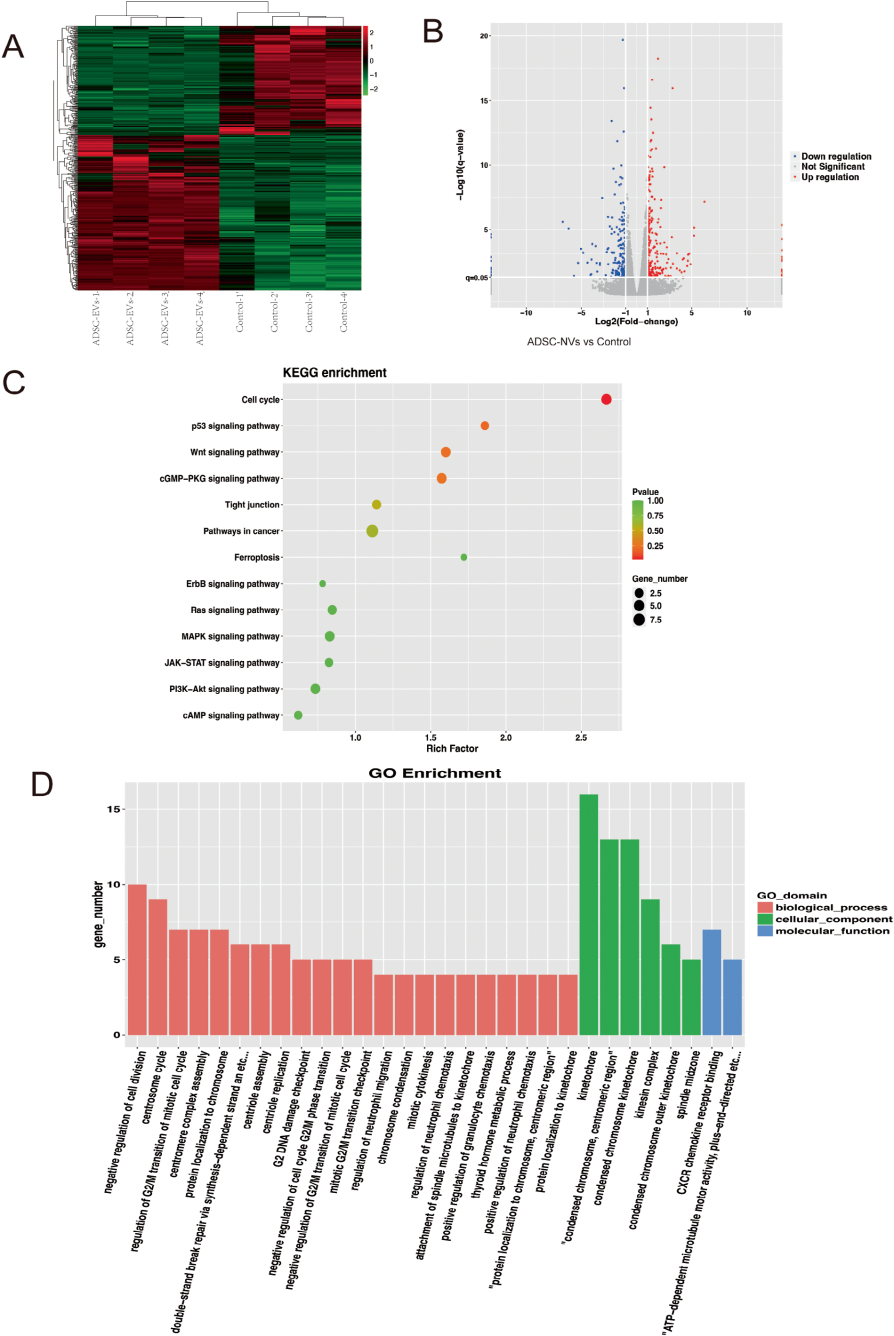
Gene expression profiling of ADSC‐NVs treated Human fibroblasts. (A) Heat map analysis of correlation; (B) Volcano plots of differentially expressed genes (DEGs) in different groups; (C) KEGG pathway analysis of differentially expressed genes; (D) GO analysis of differentially expressed genes in biological process, cellular component and molecular functions.

To further elucidate the signalling pathways through which ADSC‐NVs influence fibroblast proliferation, we collected fibroblast samples post‐treatment for transcriptional analysis. RNA sequencing (RNA‐seq) was performed to assess gene expression changes following ADSC‐NVs treatment. Initially, Pearson correlation analysis revealed consistent results across all samples, as depicted in the heat map (Figure 5A). Differential expression analysis was subsequently conducted to identify gene groups with significant differences between the control and ADSC‐NVs‐treated groups. The differential expression gene (DEG) volcano plot illustrated 431 DEGs between these groups, with 253 genes upregulated and 178 genes down‐regulated (Figure 5B). Following this, KEGG pathway analysis using the ClusterProfiler R package highlighted the enrichment of shared DEGs in pathways such as P53, Wnt, cGMP‐PKG, tight junctions and ferroptosis (Figure 5C). While other pathways such as p53 and cGMP‐PKG were also enriched, this study focused on Wnt signalling due to its established role in fibroblast proliferation and prior validation in wound models. Furthermore, Gene Ontology (GO) analysis unveiled biological processes associated with wound repair including cell proliferation, differentiation and mitosis among others (Figure 5D). These findings suggest that treatment with ADSC‐NVs enhances wound healing in a streptozotocin‐induced diabetes model.

### 3.5 ADSC‐NVs Treatment Promotes the Expression of Proliferation‐Related Molecules

**FIGURE 6:**
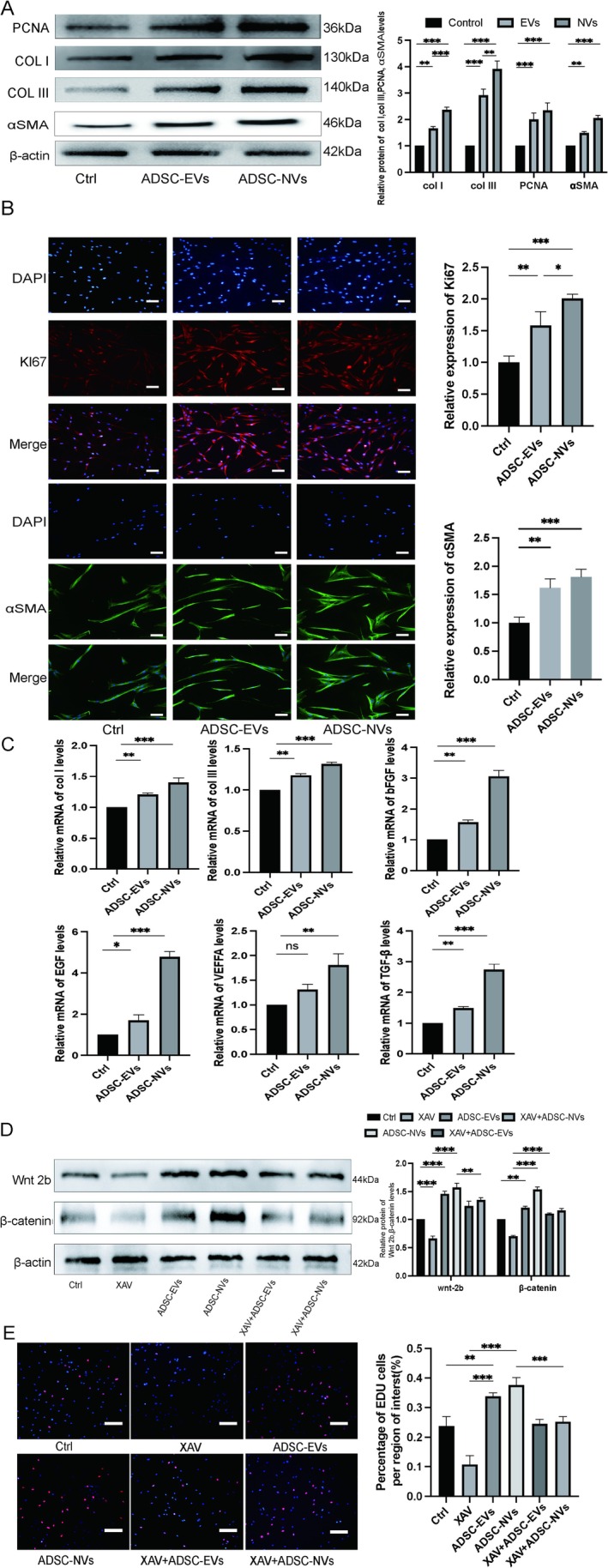
In vitro, ADSC‐NVs affect the synthesis of PCNA, collagen, α‐SMA and growth factors in HDF. (A) Analysis of protein expression levels in treated HDF; (B) Immunofluorescence analysis of Ki67, α‐SMA in HDF; (C) QRT‐PCR analysis of mRNA levels, including Col. I, Col. III, bFGF, EGF, TGF‐β; (D) Western blotting of the protein expression level of WNT2b and β‐catenin in fibroblasts treated with 50 μg/mL ADSC‐EVs, ADSC‐NVs and PBS for 36 h; (E) Edu assay showed that ADSC‐NVs‐mediated HDF proliferation was suppressed by inhibitors XAV939, compared with ADSC‐EVs. *p < 0.01, **p < 0.001, ***p < 0.0001. Scale bars, 100 μm.

Proliferation‐related proteins in fibroblasts treated with ADSC‐NVs were assessed using Western blotting and immunofluorescence. The results obtained after 24 h of treatment indicated that the expression of proliferating cell nuclear antigen (PCNA) was significantly higher in cells treated with ADSC‐NVs compared to those treated with ADSC‐EVs or untreated controls (Figure 6A). Additionally, immunofluorescence analysis revealed that the fluorescence intensity of Ki67 in cells treated with ADSC‐NVs was greater than that observed in both ADSC‐EVs‐treated and untreated cells (Figure 6B).

### 3.6 ADSC‐NVs Regulate Fibroblast Growth Factors and Extracellular Matrix Formation

The level of proteins in fibroblasts treated with ADSC‐NVs showed a notable increase (Figure 6A,B); the expression of genes COL‐I, TGF‐β, EGF and bFGF increased significantly (Figure 6C). Therefore, ADSC‐NVs regulate extracellular matrix and cytokine production in fibroblasts and have potential effects on epidermal growth and angiogenesis.

### 3.7 Regulation of WNT/β‐Catenin Signalling in Fibroblasts

The WNT/β‐catenin signalling pathway plays a critical role in promoting cell proliferation, migration and wound healing. In this study, fibroblasts were pretreated with XAV939, a specific inhibitor of the WNT/β‐catenin pathway, to investigate its regulatory effects on fibroblast behaviour. The results indicated that the levels of WNT2b and β‐catenin proteins induced by ADSC‐NVs were significantly reduced following treatment (Figure 6D). Subsequently, inhibition of WNT/β‐catenin signalling resulted in a marked decrease in ADSC‐NVs‐mediated fibroblast proliferation (Figure 6E). These findings suggest that the regulation of fibroblast proliferation and migration by ADSC‐NVs may be contingent upon the activity of the WNT/β‐catenin signalling pathway.

### 3.8 ADSC‐NVs Accelerate Skin Wound Healing in Diabetic Mice

**FIGURE 7:**
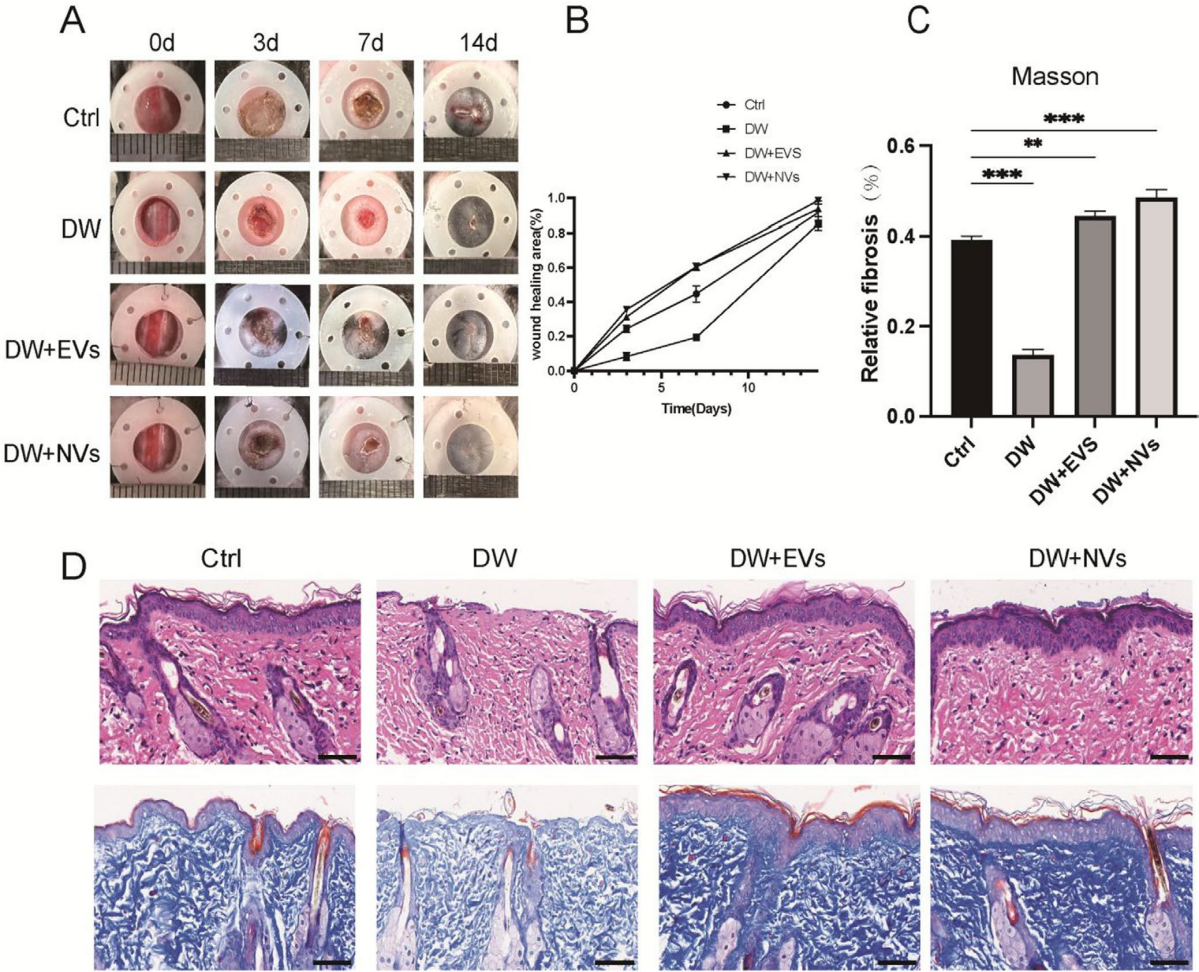
Observation of wound healing quality and velocity after operation. (A, B). After ADSC‐NVs treatment, 0, 3, 7 and 14 days were taken; (C, D). HE and Masson staining of tissue wounds 14 days after operation. *p < 0.01, **p < 0.001, ***p < 0.0001. Scale bars, 100 μm.

A diabetic wound model was established using C57BL/6 mice. Analysis of the images revealed that wound healing in the DW‐NVs, DW‐EVs and control (Ctrl) groups was significantly improved compared to the diabetic wound (DW) group. By Day 14, wounds in the DW‐NVs group were nearly completely closed. The closure rates of wounds treated with ADSC‐NVs and ADSC‐EVs were notably faster than those of untreated wounds on Days 7 and 14 (Figure 7A). Additionally, the presence of blue skin fibres in skin wounds treated with ADSC‐NVs and ADSC‐EVs indicated complete re‐epithelialization of both the epidermis and stratum corneum coverage, whereas untreated skin wounds exhibited less re‐epithelialization (Figure 7B).

### 3.9 ADSC‐NVs Modulates Inflammatory Factors, Growth Factors and Extracellular Matrix Formation in Diabetic Mice

**FIGURE 8:**
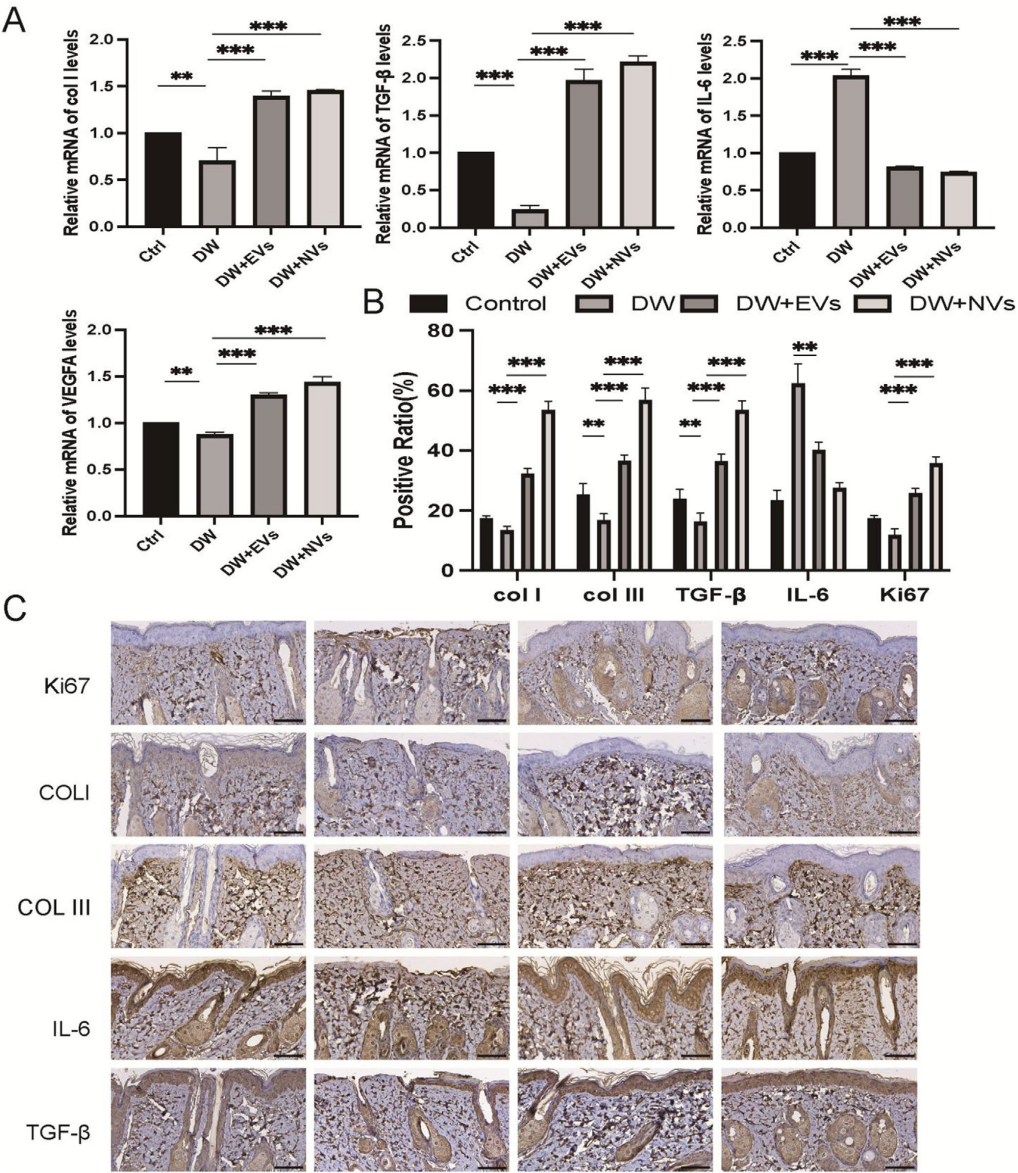
Genetic and histological analysis of wound healing in diabetes. (A) QRT‐PCR analysis of wound tissue mRNA level 14 days after operation, including COL‐I, TGF‐β, IL‐6, VEGFA; (B, C) Expression of Ki67, col. I, col. III, IL‐6, TGF‐β in wound tissue was observed 14 days after operation. *p < 0.01, **p < 0.001, ***p < 0.0001. Scale bars, 100 μm.

In the wound tissue associated with the DW‐NVs group, there was an increase in collagen and growth factor levels, including TGF‐β, COL‐I and VEGFA, within skin tissue cells; conversely, a reduction in IL‐6 expression was observed, facilitating the wound healing process (Figure 8A). This investigation focused on the impact of DW‐NVs on matrix‐related components such as Ki67, TGF‐β, COL‐I, COL‐III, and the inflammatory factor IL‐6 in diabetic wounds. Notably, compared to the diabetic wound (DW) group, those treated with DW‐NVs, DW‐EVs and control (Ctrl) groups exhibited elevated expression levels of Ki67, TGF‐β, COL‐I and COL‐III at 2 weeks post‐treatment while showing decreased IL‐6 expression (Figure 8B).

## 4 Discussion

Studies indicate that achieving the global objective of halting the rise in diabetes prevalence by 2025 is highly unlikely, with a probability of < 1% [1]. The slow wound healing and increased risk of infection in diabetic patients present significant challenges for both treatment and patient care [26, 27]. Exosomes represent a promising therapeutic avenue for tissue regeneration and have demonstrated efficacy across various tissues, including bone, cartilage, skin, tendons and nerves [28, 29, 30, 31, 32]. However, challenges such as the complex acquisition process of exosomes, low yield, and heterogeneity remain unresolved, limiting their clinical application [33]. To address these issues, efficient large‐scale production of exosomes—including engineered nanovesicles—has been proposed; engineered nanovesicles can produce significantly higher quantities than naturally secreted exosomes [21]. Some studies suggest that engineered nanovesicles may even surpass exosomes in promoting cell proliferation for regenerative purposes [34]. For instance, nanovesicles derived from bone marrow mesenchymal stem cells have been shown to facilitate osteogenesis and support bone regeneration [35], while those from adipose‐derived stem cells can enhance the vitality and proliferation of endothelial cells [36]. Based on these findings, it is hypothesised that ADSC‐NVs could potentially improve the vitality and proliferation of skin fibroblasts.

Nanovesicles are produced through a mechanism distinct from that of naturally secreted exosomes. Exosomes are meticulously regulated by cells at various states and stages [37, 38]. In contrast, nanovesicles are generated by applying an external force that pushes cells through polycarbonate membranes with varying pore sizes, resulting in the fragmentation of lipid membrane structures that subsequently self‐assemble into spherical nanovesicles [39]. The contents of these nanovesicles primarily consist of intracellular substances randomly enclosed during cell extrusion, rendering the composition of ADSC‐NVs similar to that of ADSCs [36]. This study demonstrates that ADSC‐NVs enhance fibroblast proliferation and migration by stimulating the WNT/β‐catenin signalling pathway, thereby expediting the healing process in diabetic wounds.

The WNT/β‐catenin signalling pathway plays a pivotal role in regulating cell proliferation, differentiation, migration and morphology, as well as maintaining stem cell pluripotency [40]. Increasing evidence confirms that activation of the WNT/β‐catenin signalling pathway is crucial during the proliferative phase of wound healing [41]. GO and KEGG analyses further reveal that ADSC‐NVs regulate fibroblast metabolism, differentiation and TGF‐β secretion, partially through the activation of the WNT/β‐catenin and p53 pathways. Based on these findings, we propose that ADSC‐NVs can accelerate diabetic wound healing. Furthermore, ADSC‐NVs may modulate inflammation and extracellular matrix secretion via the WNT/β‐catenin signalling pathway, contributing to enhanced diabetic wound repair. This discovery offers a promising new approach for addressing refractory diabetic wounds.

Fibroblasts play a crucial role as effector cells in the tissue repair process following injury, and their functional state significantly influences both the rate of repair and the final size of the scar [42]. In response to tissue damage, fibroblasts temporarily suspend their specialised functions and assume reparative roles, aiming to rapidly contain damage by producing a fibrillar collagen‐rich extracellular matrix (ECM) while ultimately facilitating tissue contraction [43]. Consequently, ADSC‐NVs promote fibroblast proliferation and migration. Furthermore, ADSC‐NVs enhance the synthesis of ECM components and growth factors. In vitro treatment of fibroblasts with ADSC‐NVs and ADSC‐EVs resulted in increased expression levels of PCNA and Ki67 proteins. This elevation in nuclear protein expression indicates a transition from G1 to S phase in the cell cycle, signifying that these cells are actively undergoing proliferation [44]. Transforming growth factor‐beta (TGF‐β) and basic fibroblast growth factor (bFGF) synergistically stimulate fibroblasts to induce phenotypic changes, converting them into myofibroblasts while promoting their proliferation [45]. Epidermal growth factor (EGF) further enhances both the proliferation and migration of fibroblasts [46]. In our study, TGF‐β, EGF, COL‐I, COL‐III and bFGF were significantly upregulated in fibroblasts induced by ADSC‐NVs. These findings demonstrate that ADSC‐NVs may stimulate fibroblasts to increase secretion of growth factors and ECM proteins—key elements vital for promoting healing after tissue injury.

A diabetic wound model was established using C57BL/6 mice and treated in vivo with ADSC‐NVs. Notably, compared to ADSC‐EVs, ADSC‐NVs accelerated a more efficient healing process for diabetic wounds. It is well established that collagen types I and III (COL‐I, COL‐III) are critical components secreted by fibroblasts during the wound healing process and constitute the most abundant constituents of the extracellular matrix (ECM) [47, 48]. ADSC‐NVs interact with fibroblasts to regulate ECM formation, thereby enhancing diabetic wound healing. Our findings indicate that treatment with ADSC‐NVs increased collagen levels while promoting an orderly arrangement of fibres. These results suggest that ADSC‐NVs may facilitate remodelling of the ECM in diabetic wound healing, ultimately improving scar fibrosis. We observed a significant increase in the expression levels of transforming growth factor‐beta (TGF‐β), COL‐I, COL‐III and vascular endothelial growth factor (VEGF), alongside a decrease in interleukin‐6 (IL‐6) content in diabetic mice treated with ADSC‐NVs. TGF‐β is recognised as a key growth factor associated with multiple stages of wound healing, while VEGF plays a crucial role in promoting angiogenesis during this process [49]. Compared with the existing therapy TGF‐β1 applied to diabetic wounds, ADSC‐NVs has the same wound healing time, healing rate and tissue regeneration quality [50]. Furthermore, ADSC‐NVs may enhance high‐quality skin repair through modulation of growth factor secretion and expression while concurrently reducing pro‐inflammatory factors such as IL‐6 and tumour necrosis factor‐alpha (TNF‐α). Overall, these findings suggest that ADSC‐NVs can facilitate vascularization of diabetic wounds and promote the healing process, presenting a promising strategy for achieving scarless wound repair in clinical settings.

However, the large‐scale production of ADSC‐NVs for clinical applications presents numerous challenges. It requires the integration of efficient extraction technologies, rigorous quality control standards and industrial processes that comply with Good Manufacturing Practice (GMP) requirements. Additionally, issues related to long‐term efficacy, safety, and cost‐effectiveness must be addressed to promote their widespread clinical use.

This study has several limitations. First, the relatively small sample size may limit the robustness of statistical analyses and the external applicability of the results. Second, the lack of long‐term follow‐up in the animal model prevents a comprehensive evaluation of the treatment's durability and potential late‐stage effects. Furthermore, no direct comparative analysis has been conducted between ADSC‐NVs and MSCs‐NVs for their application in diabetic refractory wounds. These factors may impose certain constraints on the interpretation of the study findings.

## 5 Conclusion

High‐yield ADSC‐NVs, which mimic extracellular vesicles, have been demonstrated in our study to enhance the healing of diabetic wounds by promoting cellular proliferation and migration through the activation of the Wnt/β‐catenin signalling pathway. Additionally, they increase the secretion of vascular growth factors and extracellular matrix components, thereby accelerating diabetic wound healing. In an in vivo model utilising STZ‐induced diabetic mice, we observed that ADSC‐NVs effectively alleviate diabetic ulcers and expedite wound healing by enhancing the secretion of multiple growth factors, collagen production, angiogenesis and reducing inflammatory mediators. The consistency between in vivo and in vitro experiments strongly supports the efficacy of ADSC‐NVs. Overall, the application of ADSC‐NVs for diabetic wound repair represents a promising new therapeutic strategy.

## Author Contributions

Tonghao Yao and Liangliang Liu were responsible for the overall design, experimental validation, and manuscript writing of the study. Yibo Miao and YingYu handled the data curation. Xinxin Li was in charge of animal experiments. Rongyao Sun, Yining Zhang and Luping Cui were in charge of the statistical analysis of experimental data. Xu Ma supervised and funded the project. All authors reviewed the manuscript.

## Disclosure

The statements, opinions and data contained in all publications are solely those of the individual author(s) and contributor(s).

## Ethics Statement

The authors pointed out that all experimental protocols of this study were approved by the Experimental Animal Ethics Committee of Harbin Medical University and the Second Affiliated Hospital of Harbin Medical University with the approval number YJSKY2023‐467, YJSDW2023‐201. Approval date: 2023‐09‐04. All methods are reported in accordance with ARRIVE guidelines (https://arriveguidelines.org).

## Consent

All the subjects and mice involved in this study were approved by the relevant institutions.

## Conflicts of Interest

The authors declare no conflicts of interest.

## Data Availability

All data generated or analysed in the course of this study are included in this published article. If you are unclear, please ask the corresponding author. Additionally, the raw date is available in a public repository at: https://www.ncbi.nlm.nih.gov/geo/query/acc.cgi?acc=GSE279804.
